# Effect of Par Frying on Composition and Texture of Breaded and Battered Catfish

**DOI:** 10.3390/foods7040046

**Published:** 2018-03-23

**Authors:** Peter J. Bechtel, John M. Bland, Kristin Woods, Jeanne M. Lea, Suzanne S. Brashear, Stephen M. Boue, Kim W. Daigle, Karen L. Bett-Garber

**Affiliations:** 1 Southern Regional Research Center, Agricultural Research Service, United States Department of Agriculture, 1100 Robert E. Lee Blvd., New Orleans, LA 70124, USA; john.bland@ars.usda.gov (J.M.B.); jeanne.lea@ars.usda.gov (J.M.L.); suzanne.brashear@ars.usda.gov (S.S.B.); steve.boue@ars.usda.gov (S.M.B.); kim.daigle@ars.usda.gov (K.W.D.); karen.bett@ars.usda.gov (K.L.B.-G.); 2 Alabama Cooperative Extension System, Auburn University, 120 Court Street, Grove Hill, AL 36451, USA; woodskl@aces.edu

**Keywords:** catfish, batters, texture, oil content

## Abstract

Catfish is often consumed as a breaded and battered fried product; however, there is increasing interest in breaded and battered baked products as a healthier alternative. Par frying can improve the texture properties of breaded and battered baked products, but there are concerns about the increase in lipid uptake from par frying. The objective of this study was to examine the effect of different batters (rice, corn, and wheat) and the effect of par frying on the composition and texture properties of baked catfish. Catfish fillets were cut strips and then coated with batters, which had similar viscosities. Half of the strips were par fried in 177 °C vegetable oil for 1 min and the other half were not par fried. Samples were baked at 177 °C for 25 min. Analysis included % batter adhesion, cooking loss, protein, lipid, ash, and moisture, plus hardness and fracture quality measured using a texture analyzer. A trained sensory panel evaluated both breading and flesh texture attributes. Results found the lipid content of par fried treatments were significantly higher for both corn and wheat batters than for non-par fried treatments. Sensory analysis indicated that the texture of the coatings in the par fried treatments were significantly greater for hardness attributes. Fillet flakiness was significantly greater in the par fried treatments and corn-based batters had moister fillet strips compared to the wheat flour batters. Texture analyzer hardness values were higher for the par fried treatments.

## 1. Introduction

Catfish production is the largest US aquaculture industry; and approximately 161 million pounds of catfish were processed in 2012 [1]. The catfish industry produces a large number of breaded and battered catfish products for deep fat frying; however, few breaded and battered products are designed for baking. The most common problem with baked battered and breaded fish products is that the texture is different (less crisp) than the fried product. One method for improving the texture properties of baked products is to par fry prior to baking [2]. An initial par frying step sets the batter and breading and results in an acceptable baked product in terms of texture. Issues associated with par frying fish products are equipment and operation expenses, and the increased percentage of calories from the oil that is adsorbed by the product. A baked product that is not par fried, but that has texture properties approaching those of a fried product, would have the potential to meet purchasing specifications for food service operations, such as school districts, health care facilities and government purchases. 

The basic operations involved in catfish processing, and further processing, have been summarized previously [3] and include batter and breading of catfish fillets. There are a number of relevant studies on the batter and breading of fish, including reports on the rheology and other physical properties of batters used with frying products [4,5,6,7,8]. Batters and breading that would alter fat uptake from frying were examined by Ang in 1993 [9], and, more recently, in a study of rice and wheat batters [10]. The texture properties of microwave-cooked breaded and battered fish were evaluated and effects of batter formulation on quality and crispness of studied microwave-cooked fish nuggets [11,12,13]. The performance effects of different frying oils on the performance used in frying of chicken and fish sticks have been reported [14]. Studies on batter ingredients to reduce oil uptake and other properties have been reported [15,16,17,18,19]. The effects of coating porosity on oil uptake have been reported [20], and the effects of different par frying temperatures on the physical characteristics of silver carp nuggets were examined [21]. Others have evaluated cooking methods on the properties of breaded black ponfret fillets and lipid oxidation of salmon and mackerel fish nuggets during frozen storage [22,23].

There are numerous examples of breaded and battered catfish products available in the market place for deep fat frying. Most battered and breaded fish products on the market, which would be baked, have undergone an initial par frying step that sets the batter and breading and results in an acceptable baked product, in terms of its texture and product appeal, when baked. The overall goal of this study was to make a battered catfish product that could be baked and have a lower percentage of oil-based calories than equivalent par fried products. The specific objectives of this study were to compare the textures and physicochemical parameters of catfish fillet strips battered with rice, corn, and wheat based flours, which would be either baked or par fried and baked. 

## 2. Materials and Methods

### 2.1. Sample Preparation

Frozen Individual Quick Frozen (IQF) catfish shank fillets (7–9 oz) were obtained from a large catfish processor. The fillets were thawed and cut into 25 to 30 g strips and then coated with either rice flour (Organic White Rice Flour, Arrowhead Mills Inc., Boulder, CO, USA), corn flour (Whole Grain Corn Flour, Bobs Red Mill Natural Foods, Milwaukie, OR, USA), or wheat flour (Unbleached All Purpose Flour, King Author Flour, Norwich, VT, USA) batters. All batters were adjusted to a similar viscosity of around 120 Rapid Visco Units (1440 Cps) by addition of water to the dry mix. The batter formulae follow: Rice flour 95.28 g, xanthan gum 0.1 g, NaCl 3 g, baking powder 1.72 g, and water; corn flour 95.28 g, NaCl 3 g, baking powder 1.72 g, and water; and wheat flour 95.28 g, NaCl 3 g, baking powder 1.72 g, and water. Individual catfish strips were coated with batter and placed on elevated racks. The dimensions of the battered and breaded catfish strips were approximately 10.1 cm in length, 2.7 cm in width and 2.1 cm in thickness. The percent batter adhering to the raw strips was determined from the weight of the individual strips before and after battering. After coating, half of the strips were par fried and the other half were not par fried. Coated strips and par fried coated strips were placed individually on trays in a −20 °C freezer, and then stored in plastic bags at −20 °C until evaluated.

The par fried treatment consisted of frying coated strips in vegetable oil at 177 °C for 1 min, which fell within the parameters of par frying [2]. After the par frying step, the strips were placed on paper towels until cool and placed in a −20 °C freezer. The baking treatment for both par fried and non-par-fried samples was 177 °C for 25 min. Cooking loss was determined for the par fried treatment by weighing battered and breaded strips before and after par frying, but before freezing. Cooking loss was determined for baked samples by weighing strips before and after baking.

### 2.2. Proximate Analysis

Moisture and ash content were determined using Association of Official Analytical Chemists (AOAC) (1990) methods #950.46 and #923.03 [24], respectively. Nitrogen content was determined using pyrolysis with a Leco TruSpec N nitrogen analyzer (Leco Co., St. Joseph, MO, USA). Protein content was calculated as 6.25 times percent N. Moisture, ash, and lipid contents were determined in duplicate and protein content were determined either in duplicate or triplicate for each replicate sample. Total lipid content was determined gravimetrically using a modification of the Folch procedure [25], using a Dionex ASE 200 accelerated solvent extractor (Dionex (UK) Ltd., Camberley, Surrey, UK) with methylene chloride at 100 °C and 1500 psi. After lipid extraction, the solvent was removed under a N_2_ gas stream at 40 °C using a TurboVap LV (Caliper Life Sciences, Waltham, MA, USA) in pre-weighed vials [26]. 

### 2.3. Color Measurement

The color of battered fillet strips were measured after baking using a Konica-Minolta Chroma meter CR-410 (Konica-Minolta Sensing, Tokyo, Japan). The colorimeter was calibrated with a white C standard plate (*Y* = 86.1, *x* = 0.3154 and *y* = 0.3229) and 2° observer angle before sample measurements. CIE Lab color space was used to record L*, a* and b* values. The L* represents the lightness of the color, where 100 represents white and zero represents black. The a* axis indicates a red shade when greater than zero (positive) and a green shade when lower than zero (negative). The b* axis indicates a yellow shade when positive and a blue shade when negative. Color values were determined for multiple locations on a strip and then an averaged value was determined for each strip. 

### 2.4. Sensory Evaluation

The breading texture attributes for this experiment were selected from those previously reported [10,27]. The attributes selected were flaky, hardness, fracturability, crispness and tooth packing [28], and are described in Table 1. The cooked fillet texture attributes used were adapted from Chambers and Robel [29] and included flaky, firmness, moisture release, fibrous, moisture retention, and cohesiveness of mass (Table 2). 

Ten panelists were trained to rate intensities of texture attributes for battered and breaded catfish. They practiced for six sessions using fish prepared in the following way: approximately 30 g of fish was fried in 177 °C vegetable oil for 3.5 min, par-fried in 177 °C oil for 1 min and then baked at 218 °C for 15 min, or baked at 218 °C for 25 min. During practice sessions, Zatarain’s Crispy Southern fish seasoning, New Orleans, LA, USA (with corn meal), Zatarain’s chicken frying mix, New Orleans, LA (with wheat flour), and Choice Batter (by CrispTek, LLC, Columbia, MD, USA) (with rice flour), were used to represent experimental formulations. One session was used to practice on breaded par-fried and breaded-only samples that were pre-frozen. The frozen samples were baked prior to presentation to panelists for evaluation. Prepared fish samples were served in warmed glass bowls placed in foam bowls to keep them warm. Samples were presented monadically to the panelists under sodium vapor lights that mask color variation. Sensory evaluations were conducted in individual booths within a climate controlled room, and scores were entered into Compusense five 5.4.15v (Guelph, ON, Canada) sensory balloting software. Unsalted soda crackers and filtered water were provided to cleanse the palate between each test sample. 

Experimental samples for sensory evaluation (see Section 2.1) were battered and individually frozen prior to the panel sessions. The par-fried samples were par-fried prior to freezing. At each experimental sensory evaluation session, two different flour treatments (with one baked sample and one par-fried sample) were presented. Both the baked sample and the par-fried then baked sample were baked at 177 °C for 25 min. Samples were served as described above. Each flour-batter treatment/cooking method combination was presented at two different panel sessions and scores were entered into Compusense five. All three flour types (corn, rice, and wheat) were paired with the other flour-batter types during one session.

### 2.5. Texture Analysis

Textural properties were measured on a Steven’s QTS Texture Analyzer (Brookfield Engineering Labs, Inc., Middleboro, MA, USA) with a 25-kg load cell using a TA-52 stainless steel shear blade. The texture was measured after samples had been baked for 25 min at 177 °C and then cooled at 21 °C for 30 min. The cooked fish was compressed in a single cycle test at 150-mm/min-test speed until a 50% deformation target was reached. Textural properties were calculated using Texture Pro2 software from Brookfield Engineering Labs, Inc. (Middleboro, MA, USA). Recorded properties included hardness, defined as the peak compression force attained in the force deformation curve, the quantity of fractures, defined as the number of occasions the load decreased by 5% prior to reaching the target value, and the sample length.

### 2.6. Statistical Analysis

The means of data were compared using an analysis of variance (ANOVA) and the mean comparison test, Tukey-Kramer adjustment to Least Squares Means, were performed in Proc Mixed using Enterprise Guide, version 5.1, (SAS, Inc., Cary, NC, USA). Significance was reported at *p* < 0.05 for all data.

## 3. Results and Discussion

### 3.1. Cooking Yield and Proximate Analysis

The average weight of the raw catfish strips used for the battering experiment was 27.5 g to 28.5 g (Table 3). After battering, the catfish strips weight increased 39.4% to 23.7%, with corn batter having the largest weight gain and wheat batter the least. A breading and batter adhesion of 25% to 30% would be reasonable for chicken or pork nuggets [2]. In this study, the fillet strips had less uniform shapes than a reformed nugget product. When the batter coated product was baked, there was a loss of weight from 27.1% to 24.5%. Par frying for 1 min at 177 °C resulted in a weight loss of 23.3% to 26.9% for the corn and wheat flour batters, respectively. Meanwhile, rice flour battered fish had a weight loss of only 15.9% in the study by Ojagh et al. [21], and they reported a product yield of approximately 80% after par frying, which was higher than the yields found in this study for the wheat and corn flour batters. 

The proximate analytical results for raw, baked, par fried (before baking) and par fried then baked samples are listed in Table 4. Within a batter treatment the par fried then baked treatments for the corn and rice batters showed significantly lower (*p* < 0.05) moisture content than coated raw, baked or par fried samples. However, for the wheat coated treatments the par fried (before baking) moisture value was not different from the coated raw value. In addition, the par fried baked moisture value was significantly different (*p* < 0.05) from the baked value for corn and rice batters. There were significant differences between the ash contents; however, ash values ranged from a low of 1.3% to a high of 2.2% on a wet weight basis. Within the baked treatments, the corn and rice battered treatments yielded significantly higher (*p* < 0.05) in moisture content than wheat baked treatments. The protein content in the par fried then baked fish was significantly higher (*p* < 0.05) than the coated raw fish with corn batter. For the wheat batter, the protein values of both par fried then baked and the baked fish were significantly higher than their raw products. This was due to the loss of moisture during cooking of the fish. Par fried and baked corn flour battered fish was significantly greater (*p* < 0.05) in protein than the raw coated fish, but the baked corn flour coated sample was not significantly different (*p* < 0.05) from the raw coated sample. The corn batter baked fish retained more moisture during baking than the wheat battered fish. The wheat baked product was significantly higher in (*p* < 0.05) protein than the corn or rice battered fish, because wheat flour (~14%) typically has a higher protein content than corn (~9%) and rice (~8%) flours. There were no significant differences (*p* < 0.05) in the protein content between the par fried than baked fish for the corn or wheat batter. The moisture of par fried breaded black pomfret values was reported to be 57%, and, after further baking, the moisture content was 56% [22]. In our study, the moisture content of par frying before baking treatments ranged from 65.8% to 72.3%; however, when the par fried product was baked, the moisture content was further reduced to 56.1% to 61.2%. The moisture and oil content of coated par fried Talang queen fish nuggets were reported to be 54.2% and 7.21%, respectively [18].

For the corn, rice and wheat flour batter treatments, the par fried then baked treatments compared to the coated raw treatments yielded significantly higher (*p* < 0.05) percent oil content. For both corn and rice flour batters, the percent oil content was significantly greater (*p* < 0.05) for the par fried then baked treatment compared to the baked treatment. The increase in percent oil between the baked and par fried then baked treatments for the corn and rice batters was 6.8% and 8.3%, respectively. These values for corn and rice batters represent increases of over 200% in the calories derived from oil between baked and par fried then baked treatments. For the wheat batter treatments, the percent oil values were not significantly different (*p* < 0.05) for the baked, par fried before baking, and par fried after baking samples; however, these treatments had significantly greater percent oil values (*p* < 0.05) than the coated raw treatment. The oil content of par fried breaded black pomfret values was reported to be 8.8%, which increased after being oven baked to 9.1% in sunflower oil and 9.5% in palm oil [22]. In our study the par fried before baking treatment had percent oil values ranging from 4.2% for the rice flour batter to 8.0% for the wheat flour batter treatment. After baking the par fried then baked, treatments had percent oil values of 9.8%, 6.9% and 10.8% for corn, wheat and rice, respectively, with rice batter having a significant increase (*p* < 0.05) between par frying and baking after par frying. 

One method to avoid increasing the oil content of battered fish products is baking without par frying the product, which is one of the treatments in this study. Another method to reduce the oil content of par fried products is to incorporate ingredients in the breading and battering that will reduce the amount of oil adsorbed on the par fried product [13,15,16,18], such as carboxyl methyl cellulose, HPMC, methylcellulose edible coatings, or whey protein concentrate films. 

### 3.2. Color Properties

Colorimetric values of the par-fried and non-par-fried fish strips are listed in Table 5. For the wheat and corn batters, the L* value was higher for baked fish than the par fried baked treatment, which indicates that the baked strips had a lighter color. However, for the rice batter, the par fried batter was lighter. Within the par fried treatments, the L* value for the rice batter was significantly higher (*p* < 0.05) than both the corn and wheat batters. For the baked treatments, the rice and wheat batters yielded significantly higher (*p* < 0.05) L* values than the corn batter. For the a* color value, the par fried wheat and corn battered fish exhibited significantly higher (*p* < 0.05) than their baked counterparts, making them more red (Table 5). For par fried fish, the a* value for the corn batter was significantly higher (*p* < 0.05) than the wheat or rice batter treatments. The corn baked fish was significantly higher (*p* < 0.05) than the rice baked, which was significantly higher than wheat baked. The b* value for par fried rice was significantly higher (*p* < 0.05) than the baked sample. However, in the baked wheat sample, b* was higher than par fried; for the corn sample, the b* values were almost equal. The corn par fried sample is significantly higher (*p* < 0.05) than the wheat or rice par fried treatments confirming an increase in yellowness. For the baked treatments, the corn battered sample was significantly higher (*p* < 0.05) than the wheat battered, which was significantly higher than the rice battered samples. 

In the study by Moradi et al. [22], they reported a decrease in the L* value and an increase in the a* and b* values when par fried black pomfret samples were baked. Others have reported that, when silver carp nuggets were par fried and the oil temperature increased, the L* value decreased and a* and b* values increased [21]. The ideal color for fried products was reported to be a light brown color [30], which would correspond to L*. a*, b* values of approximately 70, 9 and 5, respectively. The a* values of the catfish strips for all batters were lower, indicating less red in all samples.

### 3.3. Sensory Evaluation of Texture

There were significant interactions between cooking method and type of flour used in the breading formulations. The type of coating had a significant difference (*p* < 0.05) on all the breading texture attributes (Table 6). For the baked treatments, rice flour was significantly higher (*p* < 0.05) in hardness of the coating than corn and wheat flour. Hardness had a markedly high interaction between flour type and cooking method. In the par fried treatments, hardness, fracturability, crispness and tooth packing of both the corn and rice flours were significantly higher (*p* < 0.05) than the wheat flour. For tooth packing, the rice flour was significantly higher (*p* < 0.05) than the wheat flour. Greater differences were found in the par-fried treatments than in the baked treatments. 

The coatings on the par-fried baked catfish strips were significantly greater (*p* < 0.05) in intensity compared to baked coating for hardness, fracturability and crispness texture attributes (Table 6). These attributes had interaction effects that were driven by the type of flour. Studies on the sensory properties par fried fish products include evaluation of silver carp fish nuggets, par fried at different temperatures [21]. However, they did not observe significant differences (*p* < 0.05) between par frying temperatures.

The only significant difference (*p* < 0.05) between batters in the fish fillet texture was moisture retention, where the corn flour for the par fried sample was significantly higher (*p* < 0.05) than the par fried wheat and rice flour treatments (Table 7). Corn flour retained significantly (*p* < 0.05) more moisture than wheat and rice battered products. For the flaky attribute, the corn par fried sample was significantly higher (*p* < 0.05) than the rice par fried fish. The flaky attribute was significantly greater (*p* < 0.05) in the par-fried then baked fish than in the baked fish for corn batter (Table 7), which indicated that the baked fish was more difficult to flake.

### 3.4. Mechanical Texture Properties

The mechanical texture attribute of hardness was greater in the par fried then baked treatments than the baked treatments (Table 8). The par fried corn and rice coated fish samples had significantly greater (*p* < 0.05) hardness than the baked equivalents. There were no significant differences (*p* < 0.05) in hardness between wheat, corn or rice coated par fried then baked fish treatments. Neither was there a significant difference in hardness among the wheat, corn or rice coated baked fish treatments. There was no significant difference (>0.05) in the number of fractures between par-fried then baked and baked fish for each coating. However, the number of fractures were always higher in the par-fried fish. Within the baked fish samples, quantities of fractures for rice coated fish were significantly higher (*p* < 0.05) than the wheat coated fish. For par-fried then baked fish, the quantity of fractures was significantly higher (*p* < 0.05) in the rice coated fish than the wheat coated fish. Moradi et al. (2009) showed that the texture (adhesiveness, springiness, and cohesiveness) values of par fried black pomfret increased slightly when par fried samples were baked. 

## 4. Conclusions

The oil content of par fried baked products was significantly higher (*p* < 0.05) for the corn and wheat batters compared to that of comparable non par fried treatments. Sensory results indicated that the coatings on the par fried then baked catfish strips were significantly greater (*p* < 0.05) than baked coating for hardness, facturability and crispness attributes. These attributes had interaction effects that were driven by the type of batter. Fillet flakiness was significantly greater (*p* < 0.05) in the corn battered par fried then baked treatment than in the corn battered baked treatment. The corn flour battered products had moister fillet strips. Hardness values measured with a texture analyzer were higher for the par fried then baked treatment when compared to the non-par fried treatment.

## Figures and Tables

**Table 1:** Breading texture attribute lexicon.

Attribute	Definition and Reference
Hardness	The force to compress the food. 1.0 = Philadelphia Light Cream Cheese to 11.0 Uncooked carrots
Fracturability	The force with which the sample crumbles or shatters. 1.0 = Jiffy Corn Muffin to 13.0 Peanut Brittle
Crispness	The force and noise with which a product breaks rather than deforms. 3.0 = Quaker Chewy Granola Bar to 17.0 Old London Melba Toast
Toothpacking	Degree to which product sticks to the surface of the teeth. 1.0 = Raw carrots to 11.0 Cheetos

**Table 2:** Catfish fillet flesh sensory texture attributes.

Attribute	Definition and Reference
Flaky-Visual	The ease of breaking the fish into small pieces with a fork. 2.0 = Deli Turkey to 7.0 = Bumble Bee Fancy Lump crab meat
Firmness	Amount of force required to bite through the flesh when the sample is placed between molar teeth. (On hot dog, place cut surfaces between molars.) 4.0 = Hebrew National All beef hot dog to 9.0 = Cooked chicken breast
Moisture Release (Juicy Initial)	Bite with molars then evaluate the amount of liquid released when the sample is placed on tongue and pressed to the roof of the mouth. 2.0 = Oscar Meyer All beef hot dog, 11.0 = sliced orange
Fibrous	The perception of filaments or strands of muscle tissue during mastication. 2.0 = Ball Park All Beef Hot Dog, 10.0 = Cooked chicken breast
Moisture Retention (Juicy Mid-Point)	Amount of liquid observed in the mass after 5 chews with the molar teeth. 4.0 = Deli Turkey, 7.0 = Ball Park All Beef Hot Dog
Cohesiveness of Mass	The degree to which chewed sample (at 10 to 15 chews) holds together in a mass (forms a ball). 3.0 = Raw Mushroom, to 8.0 = Cooked Chicken Breast

**Table 3:** Weights of par fried and baked catfish strips coated with corn, wheat and rice batters.

	Corn		Wheat		Rice	
	Weight (g)	SD	Weight (g)	SD	Weight (g)	SD
raw	27.7	1.3	28.3	2.8	28.5	1.6
coated raw	38.6	1.8	35.0	2.8	35.9	1.7
baked	28.8	1.8	25.5	2.0	27.1	1.7
par fried	29.6	2.6	25.6	3.2	30.2	3.0
par fried + baked	24.5	3.1	17.9	1.3	25.7	3.2

Mean values with standard deviations (SD); *n* = 30 raw catfish strips; *n* = 15 raw coated catfish strips; *n* = 10 raw coated catfish strips baked; *n* = 15 raw coated catfish strips par fried; *n* = 10 raw coated catfish strips par fried and baked.

**Table 4:** Composition on a wet weight basis of baked and par fried and then baked catfish strips coated with corn, wheat and rice batters.

		% Moisture		% Ash		% Protein		% Lipids (Oil)	
		Mean	SD	Mean	SD	Mean	SD	Mean	SD
Corn	coated raw	73.2 ^A^	1.4	1.4 ^C^	0.2	13.5 ^B^	1.0	3.3 ^B^	0.2
	baked	66.7 ^Bz^	1.5	2.0 ^AB^	0.1	16.6 ^ABy^	1.1	3.0 ^By^	0.1
	par fried before baking	65.8 ^B^	3.0	1.6 ^BC^	0.1	14.9 ^AB^	2.7	7.7 ^A^	1.8
	par fried after baking	56.1 ^C^	2.5	2.1 ^A^	0.2	18.3 ^A^	1.7	9.8 ^A^	0.1
Wheat	coated raw	77.5 ^A^	1.8	1.3 ^B^	0.0	12.6 ^B^	0.8	3.6 ^B^	0.4
	baked	57.9 ^By^	1.1	2.1 ^A^	0.4	23.0 ^Az^	2.5	6.4 ^Az^	1.0
	par fried before baking	72.3 ^A^	0.4	1.6 ^AB^	0.2	15.1 ^B^	0.7	8.0 ^A^	0.7
	par fried after baking	60.3 ^B^	4.8	2.2 ^A^	0.3	20.1 ^A^	2.5	6.9 ^A^	1.5
Rice	coated raw	73.6 ^A^	1.6	1.4 ^C^	0.1	13.9 *	NA	3.6 ^B^	1.4
	baked	68.2 ^Bz^	2.2	1.8 ^A^	0.1	16.1 ^y^	1.1	2.5 ^By^	0.6
	par fried before baking	70.4 ^AB^	1.5	1.6 ^B^	0.0	16.1	1.2	4.2 ^B^	1.2
	par fried after baking	61.2 ^C^	1.8	1.9 ^A^	0.0	18.7	2.7	10.8 ^A^	2.6

Mean % *w*/*w* with standard deviation from analysis of 3 strips except the protein value for rice (*) coated raw which was a value for a single strip and par fried before bake and par fried baked which had values from 2 strips. A, B, C indicate that means (between raw, baked, par fried before bake, and par fried baked within the same batter) with different letters are significantly different (*p* < 0.05). z, y indicate that means (between baked of each batter) with different letters are significantly different (*p* < 0.05). The carbohydrate content of the stripes were not determined.

**Table 5:** Color analysis catfish strips coated with corn, wheat and rice flour batters.

		L*		a*		b*	
		Mean	SD	Mean	SD	Mean	SD
Corn	baked	60.86 ^y^	2.67	1.45 ^Bz^	0.11	34.06 ^z^	2.75
	par fried & baked	58.15 ^y^	1.13	4.7 ^Az^	1.1	33.87 ^z^	3.07
Wheat	baked	66.24 ^z^	0.97	−0.15 ^Bx^	0.18	22.15 ^y^	0.97
	par fried & baked	62.07 ^y^	4.96	0.67 ^Ay^	0.17	17.73 ^y^	2.87
Rice	baked	68.11 ^z^	3.13	0.49 ^Ay^	0.28	9.39 ^Bx^	0.62
	par fried & baked	71.85 ^z^	2.24	−0.82 ^By^	0.07	15.94 ^Ay^	0.85

Mean values with standard deviation from analysis of 10 strips. A, B indicate that means (between baked and par fried within the same batter) with different letters are significantly different (*p* < 0.05). z, y and x indicate that means (between par fried of each batter and baked of each batter) with different letters are significantly different (*p* < 0.05).

**Table 6:** Sensory properties for batter attributes.

Batter Attributes		Hardness		Fracturability		Crispness		Tooth Packing	
		Mean	SD	Mean	SD	Mean	SD	Mean	SD
Corn	baked	3.35 ^By^	1.43	2.42 ^B^	1.62	3.03 ^Bz^	1.64	5.09 ^B^	2.02
	par fried & baked	6.06 ^Az^	1.34	5.26 ^Az^	1.26	6.12 ^Az^	1.05	5.88 ^Az^	1.66
Wheat	baked	2.94 ^By^	1.03	2.26	1.83	2.13 ^By^	1.7	5.28	2.02
	par fried & baked	4.38 ^Ay^	0.94	2.71 ^y^	1.58	3.09 ^Ay^	1.53	4.34 ^y^	1.68
Rice	baked	4.61 ^Bz^	1.64	3.06 ^B^	1.76	3.17 ^Bz^	1.71	5.47 ^B^	1.55
	par fried & baked	6.16 ^Az^	1.52	4.47 ^Az^	1.3	5.53 ^Az^	1.54	6.29 ^Az^	1.43

A, B indicate that means (between par fried and baked of the same batter) with different letters are significantly different (*p* < 0.05); z, y indicate that means (between par fried of each batter and baked of each batter) with different letters are significantly different (*p* < 0.05).

**Table 7:** Sensory properties for fish flesh texture attributes.

		Flaky		Firmness		Moisture Release		Fibrous		Moisture Retention		Cohesiveness of Mass	
		Mean	SD	Mean	SD	Mean	SD	Mean	SD	Mean	SD	Mean	SD
Corn	baked	3.85 ^B^	1.46	3.85	1.41	5.03	1.64	4.79	1.41	5.15	1.49	5.35	1.26
	Par fry & Baked	5.35 ^Az^	1.48	3.79	1.52	5.41	1.53	4.82	1.42	5.65 ^z^	1.44	5.74	1.58
Wheat	baked	4.28	1.79	4.56	1.91	4.83	1.21	4.69	1.09	4.69	0.93	5.28	1.09
	par fry & baked	4.68 ^zy^	1.54	3.82	1.07	4.76	1.67	4.34	1.3	4.68 ^y^	1.53	5.5	1.22
Rice	baked	3.81	1.5	4.51	1.79	4.58	1.63	4.83	1.21	4.72	1.49	5.03	1.02
	par fry & baked	4.28 ^y^	1.22	4.25	1.92	4.61	1.54	4.89	1.49	4.66 ^y^	1.36	4.88	1.33

A, B indicate that means (between par fry and baked of the same batter) with different letters are significantly different based on Tukey-Kramer adjustment to the least square mean test. z, y indicate that means (between par fry of each batter and baked of each batter) with different letters are significantly different (*p* < 0.05) based on Tukey-Kramer adjustment to the least square mean test.

**Table 8:** Mechanical texture properties of catfish strips coated with corn, wheat and rice batters.

		Hardness		Quant Fract	
		Mean	SD	Mean	SD
Corn	baked	203.7 ^B^	30.2	67.6 ^Azy^	10.3
	par fried & baked	255.5 ^A^	66.2	73.3 ^Azy^	10.5
Wheat	baked	182.6 ^A^	77.6	57.2 ^Ay^	15.9
	par fried & baked	254.1 ^A^	87.8	64.3 ^Ay^	13.9
Rice	baked	200.1 ^B^	38.9	71.6 ^Az^	8.6
	par fried & baked	310.3 ^A^	60.4	79.5 ^Az^	14.6

Mean values of 10 strips per treatment with standard deviations. Quant Fract is the quantity of fractures (see Section 2). A, B indicate that means (between par fried then baked and baked of the same batter) with different letters are significantly different (*p* < 0.05). z, y indicate that means (between par fried then baked or each batter and baked for each batter) with different letters are significantly different (*p* < 0.05).
